# Retrospective Observational Study on Assessing Sitagliptin and Dapagliflozin as a Fixed-Dose Combination in the Indian Population With Type 2 Diabetes Mellitus: The SIDAXA Study

**DOI:** 10.7759/cureus.60815

**Published:** 2024-05-21

**Authors:** Manoj Chawla, Dharmarajan Panneerselvam, Abhay Gundgurthy, Sanjay Sud, Ravi Alamchandani, Pankaj Aneja, Rathish Nair, Krishnaprasad R Korukonda

**Affiliations:** 1 Department of Diabetes and Endocrinology, Lina Diabetes Care Centre, Mumbai, IND; 2 Institute of Diabetology, Madras Medical College and Government General Hospital, Chennai, IND; 3 Department of Diabetes and Endocrinology, Sanjeevani Clinic, Bangalore, IND; 4 Department of Diabetes and Endocrinology, Doctor Sud's Clinic, Hooghly, IND; 5 Department of Internal Medicine, Shivansh Hospital, Ahmedabad, IND; 6 Department of Internal Medicine, Naveda Healthcare Centre, New Delhi, IND; 7 Medical Strategic Affairs, Torrent Pharmaceuticals Ltd., Ahmedabad, IND; 8 Medical Affairs, Torrent Pharmaceuticals Ltd., Ahmedabad, IND

**Keywords:** glycated haemoglobin (hba1c), fasting plasma glucose (fpg), postprandial blood glucose (ppbg), type-2 diabetes mellitus, dapagliflozin, sitagliptin, fixed-dose combination

## Abstract

Introduction

Type 2 diabetes mellitus (T2DM), a prevalent chronic metabolic disorder, necessitates multifaceted treatment approaches. Emerging studies highlight the cardiovascular advantages of sodium-glucose transport protein 2 (SGLT2) and dipeptidyl peptidase 4 (DPP-4) inhibitors in T2DM. This investigation delves into the synergistic effects of the fixed-dose combination (FDC) of sitagliptin and dapagliflozin, offering insights into its safety and efficacy for the Indian population.

Methods

This real-world, retrospective, observational study spanned 328 cases across 111 Indian centres, evaluating the safety, efficacy, and clinical utilization of the sitagliptin and dapagliflozin FDC in T2DM patients after obtaining ethical approval. Assessments at baseline, week four, and week 12 encompassed hemoglobin A1C (HbA1C), fasting plasma glucose (FPG), postprandial blood glucose (PPBG), low-density lipoprotein cholesterol (LDL-C), systolic blood pressure (SBP), diastolic blood pressure (DBP), and weight change. The statistical analysis was done using Statistical Package for Social Sciences (SPSS) version 29.0.1.0(171) (IBM Corp., Armonk, NY, USA) with a significance level p<0.05.

Results

Study participants [mean age: 51.14±5.55 years, 77.74% (n=255) males, 22.26% (n=73) females] exhibited prevalent risk factors like sedentary lifestyle (n=167, 50.91%) and smoking (n=147, 44.82%). Comorbidities included hypertension (n=235, 71.65%) and dyslipidaemia (n=139, 42.38%). Metformin (n=282, 85.98%) and sulfonylurea (n=134, 40.85%) were commonly prescribed concomitant oral antidiabetic agents (OADs). FDC administration significantly reduced HbA1c by 1.05 ± 0.83% (p < 0.0001) at week 12. FPG and PPBG showed significant reductions of 22.98 ± 22.23 mg/dL (p < 0.0001), 165.50 ± 37.02 mg/dL and 40.94 ± 36.04 mg/dL (p < 0.0001) at four weeks respectively. By week 12, significant reductions were noted in SBP (14.61±13.98mmHg reduction, p-value <0.0001), DBP (7.80±8.45mmHg reduction, p-value <0.0001), and LDL-C levels (18.14±23.95 mg/dL reduction, p-value <0.0001). In patients with established cardiovascular disease, there was reduction in HbA1c levels by 1.02 ± 0.63% after 12 weeks, with FPG decreasing by 54.52 ± 32.67 mg/dL and PPBG decreasing by 88.73 ± 44.90 mg/dL. Treatment-emergent adverse events included headache, changes in micturition, genital mycotic infection, and nausea and diarrhoea which were mild, transient, and necessitated no treatment discontinuation.

Conclusion

The FDC of sitagliptin and dapagliflozin significantly improved glycaemic control and lipid profiles in T2DM patients, particularly those with coronary artery disease. It demonstrated a favourable safety profile in the Indian population, signifying its potential as an effective and well-tolerated therapeutic option in patients with established cardiovascular disease.

## Introduction

Type 2 diabetes mellitus (T2DM) presents a significant global health challenge, affecting millions worldwide, with India notably bearing a substantial burden as the diabetes capital of the world, hosting an estimated 77 million individuals living with diabetes. The unique characteristics of the Indian population, including a high prevalence of comorbid conditions like hypertension, dyslipidaemia, and obesity, underscore the critical need for effective T2DM management strategies tailored to this demographic. Uncontrolled diabetes poses severe long-term complications such as cardiovascular disease, retinopathy, neuropathy, and nephropathy, emphasizing the importance of timely and comprehensive management to prevent adverse outcomes and enhance quality of life. As the prevalence of T2DM continues to rise globally, there is a growing emphasis on personalized treatment approaches that not only target glycaemic control but also address individual risk profiles and comorbidities to optimize patient outcomes [1-4].

Type 2 diabetes management involves dealing with a complex landscape to achieve optimal glycaemic control. The strategic combination of therapeutic agents with complementary mechanisms of action becomes crucial in this intricate landscape. Current oral antidiabetic agents (OADs) predominantly work by enhancing insulin secretion or tissue responsiveness, intricately tied to diminishing pancreatic β-cell efficacy. As β-cell function deteriorates progressively, a transition to multiple agents is often required for target haemoglobin A1c (HbA1c) levels. However, prevalent OADs bring undesirable side effects like hypoglycaemia and weight gain, acting as barriers to dual glycaemic and weight control [5-7].

The combination therapy of sitagliptin and dapagliflozin in the treatment of type 2 diabetes, as explored in the SIDAXA Study, aligns with guidelines from the Research Society for the Study of Diabetes in India (RSSDI) and the American Diabetes Association (ADA). The RSSDI-Endocrine Society of India (ESI) Clinical Practice Recommendations emphasize the importance of glycaemic efficacy, safety profiles, weight impact, and hypoglycaemia risk in treatment decisions. Additionally, a randomized clinical trial comparing sitagliptin with dapagliflozin demonstrated non-inferiority and superiority of sitagliptin in lowering HbA1c levels. Furthermore, the Consensus Statement on Diabetes and Heart Failure highlights the evolving role of dapagliflozin in managing heart failure in patients with type 2 diabetes. These guidelines collectively support the use of sitagliptin and dapagliflozin combination therapy for managing type 2 diabetes effectively [8-10].

Sitagliptin competitively inhibits dipeptidyl peptidase 4 (DPP-4), preventing the breakdown of incretins glucagon‐like peptide‐1 (GLP‐1) and gastric inhibitory polypeptide (GIP), which leads to increased insulin secretion and decreased glucagon release, thereby lowering blood glucose levels. On the other hand, dapagliflozin, a sodium-glucose cotransporter-2 (SGLT2) inhibitor, reduces hyperglycaemia independently of insulin by selectively inhibiting the SGLT2 transporter, promoting urinary glucose excretion. The combination of sitagliptin and dapagliflozin offers a synergistic effect in managing type 2 diabetes. Clinical studies have shown that adding dapagliflozin to sitagliptin therapy provides additional clinical benefits with good tolerability, improving glycaemic control and reducing body weight without causing hypoglycaemia. This combination targets multiple pathways in type 2 diabetes management, offering extra-glycaemic benefits such as weight and blood pressure reduction, making it a valuable early treatment option. The complementary actions of these drugs make their combination a rational choice for patients with type 2 diabetes [3,9,11-13].

Combining sitagliptin and dapagliflozin in a fixed-dose combination (FDC) for management of type 2 diabetes offers several advantages. This combination can enhance patient adherence by simplifying dosing regimens, reducing the pill burden, and potentially improving treatment outcomes. FDCs like sitagliptin plus dapagliflozin can provide effective glycaemic control, reduce costs, and increase patient compliance, contributing to improved efficacy in managing type 2 diabetes. The use of FDCs is particularly beneficial for patients newly diagnosed with type 2 diabetes [14-16].

This study delves into the clinical intricacies of type 2 diabetes mellitus cases necessitating a combination of sitagliptin and dapagliflozin, specifically those exhibiting one or more risk factors or established coronary artery disease (CAD) conditions stable for more than three months post-hospitalization. Executed as a meticulous, multicentre study encompassing 328 participants, the Post Approval Concurrent Observational Study to Assess Safety, Clinical Utilization, and Effectiveness of Sitagliptin & Dapagliflozin Combination Therapy in the Treatment of Type 2 Diabetes for Extra Glycaemic Advantages (SIDAXA Study) unfolded across diverse outpatient settings in India [12,17].

This comprehensive investigation, committed to unravelling the intricate interplay of therapeutic modalities, seeks to contribute substantively to the evolving narrative of type 2 diabetes management. As we navigate this intricate realm of metabolic health, the study aims not only to shed light on clinical nuances and outcomes associated with dual therapy but also to underscore its potential as a pivotal player in the ongoing refinement of type 2 diabetes treatment paradigms.

## Materials and methods

Ethical conduct of the study

The study adhered to ethical principles outlined in the Declaration of Helsinki, Ethical Guidelines for Biomedical Research on Human Patients, Indian Council of Medical Research New Delhi 2006, GCP, New Drugs and Clinical Trials Rules 2019, and local regulatory requirements. Approved by the Sangini Hospital Ethics Committee (EC Registration number: IORG0007258) and registered with the clinical trials registry of India (CTRI/2023/06/053828), the study ensured all enrolled subjects received a Patient Information Sheet detailing essential trial information, including study description, purpose, treatment options, and patient rights.

Study design

In this retrospective observational study encompassing 111 centres across India, the objective was to assess the efficacy and safety of the FDC of vildagliptin and dapagliflozin in managing patients diagnosed with T2DM. The primary endpoints included change in HbA1c at 12 weeks, and fasting plasma glucose (FPG) and postprandial blood glucose (PPBG) at four and 12 weeks. Secondary endpoints consisted of incidence of headache, urination, yeast infections and GI disorders as well as change in systolic blood pressure (SBP) and diastolic blood pressure (DBP), low-density lipoprotein cholesterol (LDL-C), and weight at 12 weeks. The dataset included data from 328 patients, collected from centres across the country.

The study population consisted of both male and female individuals aged between 18 and 59 years, all diagnosed with T2DM and exhibiting uncontrolled HbA1c levels surpassing 7%. Inclusion criteria involved patients necessitating dapagliflozin/sitagliptin as a fixed-dose combination, in addition to their current therapeutic regimen. Additionally, participants with one or more risk factors or stable CAD for more than three months post-hospitalization were considered. A crucial condition for inclusion was the commitment to a 12-week follow-up at the same clinical centre.

Conversely, exclusion criteria were defined to ensure the relevance and safety of the study. Excluded were patients with hypersensitivity to SGLT2 inhibitors (SGLT2i) or metformin extended-release formulation, those with unstable or untreated CAD, and individuals with chronic kidney disease (CKD) and an estimated glomerular filtration rate (eGFR) below 45 ml/min. Further exclusions comprised patients with hepatic enzyme elevation exceeding three times the upper limit of normal, a history of diabetic ketoacidosis (DKA), or prior use of specific SGLT2 inhibitors.

The exclusion criteria also extended to pregnant or breastfeeding women, individuals with type 1 diabetes, sexually active women of childbearing age not practicing accepted birth control methods, and those with urinary tract infections or acute pyelonephritis in the 30 days preceding study entry. By meticulously defining the study population and excluding potential confounding factors, the methodology aimed to provide a comprehensive understanding of the real-world efficacy and safety considerations associated with the prescribed FDC regimen for T2DM patients.

Study procedure

The study involved 328 patients, who the inclusion and exclusion criteria and adhered to a 12-week follow-up period. The observational nature of the study negated the need for written informed consent, reflecting the real-world setting.

The study consists of multiple key visits. The Baseline Visit (Visit 1) coincided with the administration of the study drug. Before any assessments, patients received comprehensive information about the study and potential associated risks. Visit 1 encompassed inclusion/exclusion criteria assessment, demographic profiling, medical and treatment history, and evaluations of HbA1c, LDL-C, FPG, PPBG, SBP, DBP, and body weight. Subsequent visits at Week 4 (Visit 2) and Week 12 (Visit 3) involved the same efficacy evaluations along with safety evaluations.

Study drug

The studied drug was an FDC of sitagliptin and dapagliflozin, manufactured by Torrent Pharmaceuticals Ltd. (Ahmedabad, India). This tablet offered two strength variations: sitagliptin 100mg/dapagliflozin 10mg and sitagliptin 100mg/dapagliflozin 5mg. The dosage form involved initiating treatment with sitagliptin 100mg and dapagliflozin 5mg, with escalation to sitagliptin 100mg and dapagliflozin 10mg recommended for subsequent therapy. Administration was oral, and patients were directed by physicians based on individualized needs.

Data evaluation

Data were accurately recorded in paper case report forms (CRF) and cross-verified with source documents. After paper CRF data entry, a data lock activity was conducted, followed by a thorough data quality check. Upon ensuring data quality, an Excel-based database was transferred to statistical software for comprehensive analysis.

Statistical methods

The analytical dataset comprised a full analysis set for statistical evaluations, considering data from all individuals in the study. Subgroup analyses were further conducted, exploring specific variables related to demographics, medical history, risk factors, comorbidity, and treatment therapy for T2DM. Descriptive statistics and statistical tests, such as Student's T and Fischer's Exact Test, were employed for data presentation and significance assessment using Statistical Package for Social Sciences (SPSS) version 29.0.1.0(171) (IBM Corp., Armonk, NY, USA), with a p-value <0.05 considered clinically significant in the two-tailed test.

## Results

Patient demographics and baseline characteristics

**Figure 1:**
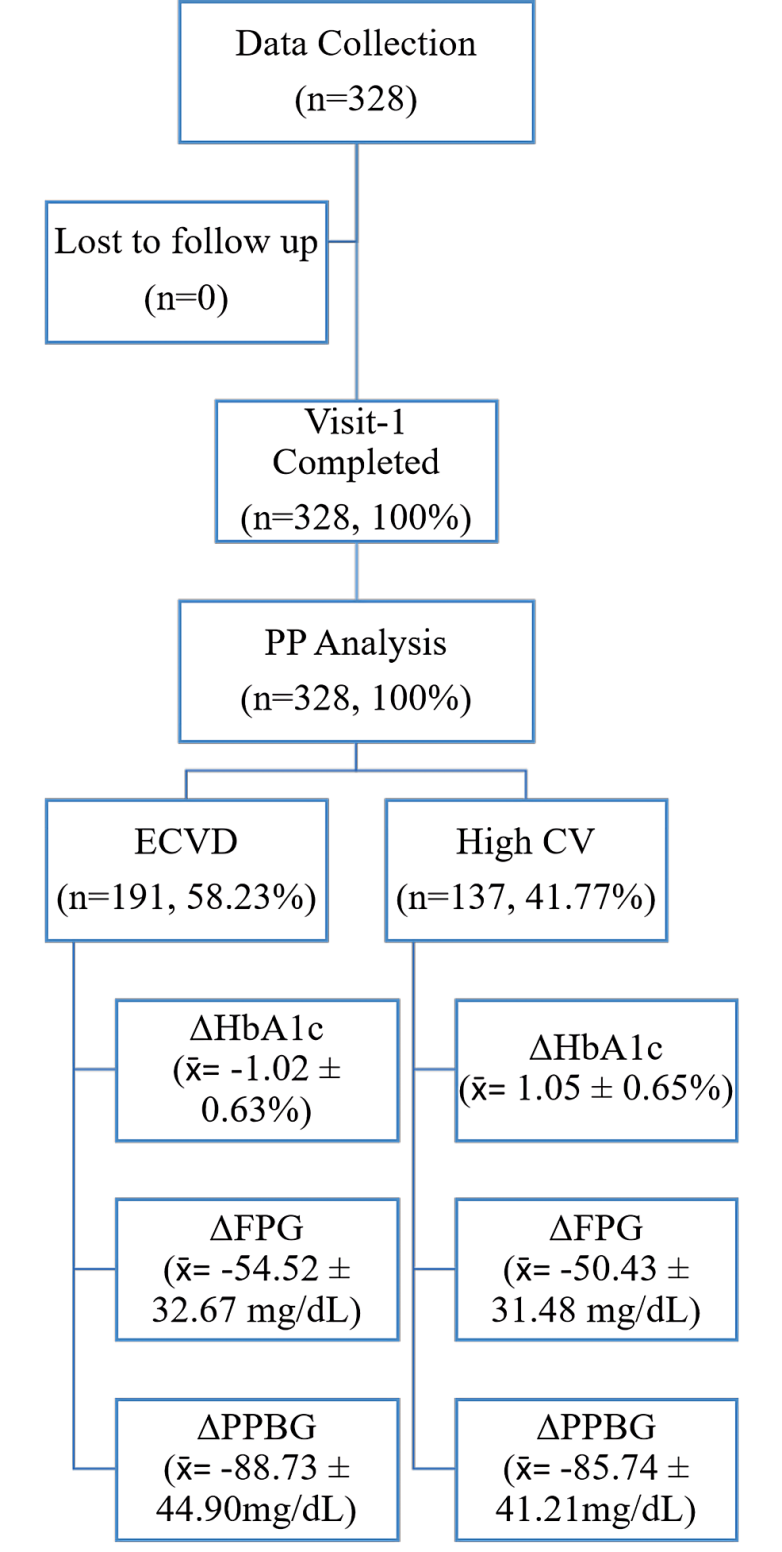
Disposition of the study participants across the study duration. N=328 (overall population) x̄: Mean ± Standard Deviation; ECVD: Established Cardiovascular Disease; CV: Cardiovascular; HbA1c: Glycated Hemoglobin; FPG: Fasting Plasma Glucose; PPBG: Post-Prandial Blood Glucose

This real-world, multicentre study involved 328 patients across 111 centres in India (Figure 1). With a mean age of 51.14 ± 5.55 years, the cohort comprised 77.74% (n=255) males and 22.26% (n=73) females, demonstrating an average BMI of 28.21 ± 3.67 kg/m².

Male participants had a mean height of 164.50 ± 7.30 cm and a mean weight of 75.36 ± 8.30 kg, while females exhibited 159.30 ± 9.87 cm and 73.41 ± 9.35 kg respectively. Lifestyle factors included 50.91% (n=167) with a sedentary lifestyle, 44.82% (n=147) smokers, 40.24% (n=132) alcohol consumers, and 18.60% (n=61) classified as obese.

Medical history revealed prevalent conditions, including myocardial infarction (n=191, 58.23%), heart failure (n=117, 35.67%), and ischemic heart disease (n=69, 21.04%). Hypertension was the most common comorbidity (n=235, 71.65%), followed by dyslipidaemia (n=139, 42.38%). Coronary artery disease, chronic kidney disease, left ventricular dysfunction, and stroke were present in 27.44% (n=90), 13.11% (n=43), 12.50% (n=41), and 7.32% (n=24), respectively, with a family history of diabetes noted in 70.73% (n=232) of cases (Table 1).

**Table 1:** Baseline Characteristics of Study Population N=number of subjects

Variables			N	Percentage (N=328)
Demographics	Age (years) (mean±SD)		51.14 ± 5.55	
	Height (cm) (mean±SD)		163.3 ± 8.21	
	Weight (kg) (mean±SD)		74.92 ± 8.57	
	BMI (kg/m2) (mean±SD)		28.21 ± 3.67	
	Gender (N, %)	Male	255	77.74%
		Female	73	22.26%
Identified risk factors	Sedentary lifestyle		167	50.91%
	Smoking		147	44.82%
	Alcoholism		132	40.24%
	Obesity		61	18.60%
Patient with history of established ASCVD (Atherosclerotic cardiovascular disease)	Myocardial Infarction		191	58.23%
	Heart Failure		117	35.67%
	Ischemic Heart Disease		69	21.04%
Comorbid conditions present	Hypertension		235	71.65%
	Dyslipidaemia		139	42.38%
	Coronary Artery Disease		90	27.44%
	Chronic Kidney Disease		43	13.11%
	Left Ventricular Dysfunction		41	12.50%
	Stroke		24	7.32%
	Peripheral Arterial Disease		10	3.05%
Family history of Diabetes	Yes		232	70.73%
	No		96	29.27%
Ongoing treatment for diabetes	Metformin		282	85.98%
	Sulfonylurea		134	40.85%
	α-glucosidase inhibitor		57	17.38%
	Gliclazide		53	16.16%
	Insulin		18	5.49%
	Pioglitazone		16	4.88%
	GLP1RA		14	4.27%
Other Co-prescribed agents for comorbid condition(s)	Anti-hypertensive agents		206	62.80%
	Lipid lowering drugs		134	40.85%
	Anti-platelet agents		122	37.20%
	Anti-Obesity		55	16.77%
	Anti-thrombotic agents		26	7.93%
Prescribed dose	Sitagliptin 100 mg & Dapagliflozin 10 mg		313	95.43%
	Sitagliptin 100 mg & Dapagliflozin 5 mg		15	4.57%

Concomitant treatment details indicated prevalent metformin usage (n=282, 85.98%), followed by sulfonylurea (n=134, 40.85%) and insulin (n=18, 5.49%). The primary prescription for diabetes management was sitagliptin 100mg and dapagliflozin 10mg (n=313, 95.43%). Additionally, patients received prescriptions for comorbid conditions, including anti-hypertensive agents (n=206, 62.80%), lipid-lowering drugs (n=134, 40.85%), anti-platelet drugs (n=122, 37.20%), anti-obesity drugs (n=55, 16.77%), and anti-thrombotic agents (n=26, 7.93%) (Table 1).

Primary endpoint results

The FDC of sitagliptin and dapagliflozin exhibited noteworthy efficacy in the management of T2DM, as demonstrated by the primary endpoints assessed in this comprehensive study.

**Figure 2:**
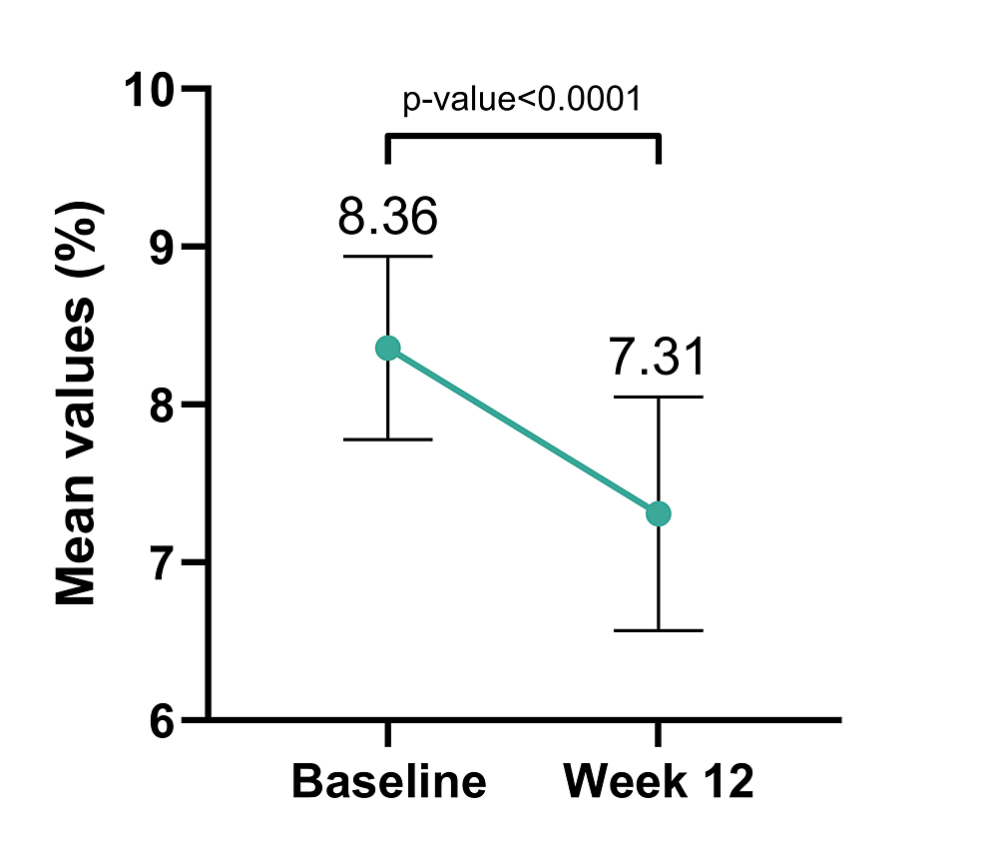
Change in Mean HbA1c (%) from Baseline to Week 12 N=328 (overall population). Values at 12 weeks are significant at p<0.01 (derived using paired t-test). HbA1c: glycated hemoglobin

HbA1c levels witnessed a substantial reduction after 12 weeks of FDC administration. The mean baseline HbA1c, initially recorded at 8.36 ± 0.58%, demonstrated a significant decrease to 7.31 ± 0.74% (1.05±0.83 reduction, p-value <0.0001), emphasizing the considerable improvement in glycaemic control achieved with FDC usage (Figure 2). Sitagliptin plus dapagliflozin as an add-on to metformin (1.5 gm/day or equivalent) showed reduction in HbA1c, PPBG and FPG.

**Figure 3:**
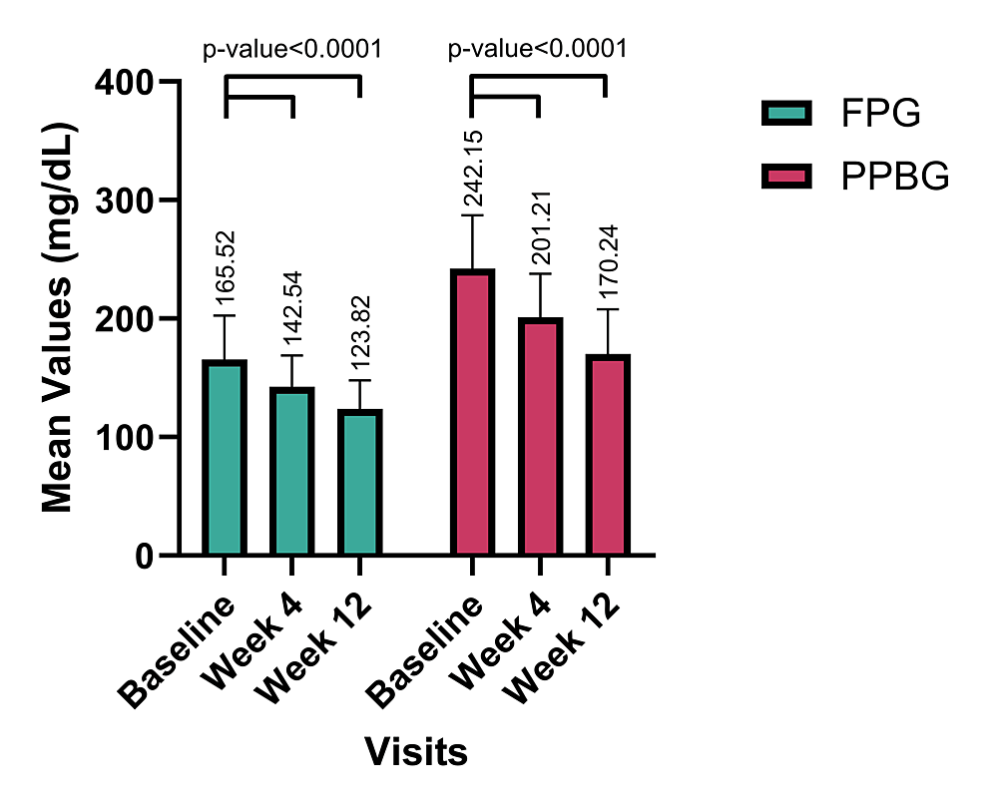
Change in Mean FPG (mg/dL) and PPBG (mg/dL) from Baseline to Week 12 N=328 (overall population). Values at four and 12 weeks are significant at p<0.01 (derived using paired t-test). FBG: Fasting Plasma Glucose; PPBG: Post-Prandial Blood Glucose

The impact of FDC on glucose control was further evident in the reduction of FPG and PPBG levels. At the four-week mark, FPG values decreased from a baseline of 165.52 ± 37.02 mg/dL to 142.54 ± 26.20 mg/dL (22.98±22.23 reduction, p-value <0.0001). Similarly, PPBG values decreased from 242.15 ± 45.17 mg/dL to 201.21 ± 36.65 mg/dL (40.94±36.04 reduction, p-value <0.0001). After eight weeks of FDC use, the FPG further reduced to 123.82 ± 23.91 mg/dL (41.70±32.96 reduction, p-value <0.0001), and PPBG reduced to 170.24 ± 37.59 mg/dL (71.91±49.87 reduction, p-value <0.0001), indicating a substantial improvement in glucose control (Figure 3).

Secondary endpoint results

Angiotensin receptor blockers (ARBs) and calcium channel blockers (CCBs) with or without β-blockers and diuretics were concomitant medications prescribed among study population.

Lipid profile improvements were observed with FDC administration in the reduction of LDL-C levels. The mean baseline LDL-C, initially recorded at 121.40 ± 28.57%, demonstrated a significant decrease to 103.20 ± 28.34% (18.14±23.95 mg/dL reduction, p-value <0.0001) at the 12-week mark, highlighting the efficacy of FDC in positively impacting lipid profiles.

Notably, FDC showcased significant and sustained improvement in blood pressure control. The baseline SBP and DBP values were 147.00 ± 16.03 mmHg and 90.31 ± 8.34 mmHg, respectively. By week 12, significant reductions were noted, with SBP at 132.40 ± 10.14 mmHg (14.61±13.98 reduction, p-value <0.0001) and DBP at 82.52 ± 8.07 mmHg (7.80±8.45 reduction, p-value <0.0001). In addition to glycaemic and cardiovascular benefits, FDC exhibited a significant impact on weight management. The mean baseline weight of 74.92 ± 8.57 kg demonstrated a statistically significant decrease to 72.76 ± 7.89 kg (2.17 ± 8.22 kg reduction, p-value <0.0001) at week 12, highlighting an additional therapeutic benefit (Table 2).

**Table 2:** Change in the Mean Blood Pressure, Weight, and LDL-C N=328 (overall population). Values at four and 12 weeks are significant at p<0.05 (derived using paired t-test). LDL-C: low-density lipoprotein cholesterol, SBP: systolic blood pressure, DBP: diastolic blood pressure

n=328	Baseline	Week 4	Week 12	Baseline	Week 4	Week 12
	SBP (mmHg)			DBP (mmHg)		
Mean	147.00	139.40	132.40	90.31	85.31	82.52
Std Dev	16.03	13.89	10.14	8.34	7.59	8.07
P-value	-	<0.0001	<0.0001	-	<0.0001	<0.0001
	Weight (kg)			LDL-C (mg/dL)		
Mean	74.92	73.86	72.76	121.40	-	103.20
Std Dev	8.57	8.03	7.89	28.57	-	28.34
P-value	-	<0.05	<0.0001	-	-	<0.0001

In summary, the primary and secondary endpoint results collectively underscore the efficacy of the FDC of sitagliptin and dapagliflozin in achieving comprehensive glycaemic control, lipid profile improvement, blood pressure reduction, and weight management in patients with T2DM.

In terms of Treatment-Emergent Adverse Events (TEAE), adverse drug reactions were observed in the study population. Notably, 6.71% (n=22) of patients reported experiencing headaches, while changes in micturition were noted in 5.49% (n=18) of individuals. Genital mycotic infections were reported by 2.13% (n=7) of patients, and nausea and diarrhoea were observed in 1.83% (n=6) of the study participants. These findings provide insights into the safety profile of the treatment, highlighting specific adverse reactions that were monitored during the course of the study.

Responder rate analysis

In the responder rate analysis among patients with comorbidities, significant improvements in achieving HbA1c levels below 7% were observed. In patients with hypertension, out of 221 patients with HbA1c between 7-9% at baseline, 78 (33.19%) achieved HbA1c below 7% at week 12. In patients with dyslipidaemia, 55 (39.57%) out of 127 achieved HbA1c below 7% at week 12. Among patients with CAD, 18 (20.00%) achieved HbA1c below 7%. In patients with CKD, 18 (41.86%) out of 40 achieved HbA1c below 7% at week 12. Among patients with left ventricular dysfunction (LVD), 12 (29.27%) achieved HbA1c below 7%.

Sub-group analyses

In patients with high cardiovascular (CV) risk (>1 risk factors), significant reductions were observed in HbA1c levels, with reductions of 0.58±0.39% after four weeks and 1.05±0.65% after 12 weeks. Additionally, FPG and PPBG decreased by 50.43±31.48 mg/dL and 85.74±41.21 mg/dL, respectively.

Similar significant reductions were noted in patients with end organ damage. In patients with LVD, HbA1c decreased by 0.64±0.58% at week four and 1.16±0.60% at week 12, while FPG and PPBG decreased by 54.56±38.88 mg/dL and 87.51±64.91 mg/dL, respectively. In patients with CKD, HbA1c decreased by 0.69±0.57% at week four and 1.25±0.85% at week 12, with FPG and PPBG decreasing by 29.05±24.52 mg/dL and 60.95±45.55 mg/dL, respectively.

Furthermore, in patients with established CVD (CAD patients), HbA1c showed reductions of 0.57±0.36% after four weeks and 1.02±0.63% after 12 weeks, with FPG and PPBG decreasing by 54.52±32.67 mg/dL and 88.73±44.90 mg/dL, respectively (Table 3).

**Table 3:** Change from Baseline in Subsets at Week 4 and Week 12 n=number of subjects. Values at four and 12 weeks are significant at p<0.01 (derived using paired t-test). CV: Cardiovascular; LVD: Left Ventricular Dysfunction; CKD: Chronic Kidney Disease; CAD: Coronary Artery Disease

	High CV risk (>1 risk factors) (n=137)			LVD (n=41)			CKD (n=43)			CAD (n=90)		
	Base-line	Week 4	Week 12	Base-line	Week 4	Week 12	Base-line	Week 4	Week 12	Base-line	Week 4	Week 12
HbA1c (%)												
Mean	8.48	7.90	7.43	8.47	7.82	7.31	8.37	7.68	7.12	8.41	7.85	7.40
Std Dev	0.50	0.53	0.69	0.83	1.03	0.85	0.51	0.61	0.72	0.54	0.64	0.66
P-value		<0.0001	<0.0001		<0.0001	<0.0001		<0.0001	<0.0001		<0.0001	<0.0001
FPG (mg/dL)												
Mean	172.62	146.10	122.19	178.51	149.68	123.95	156.79	139.16	127.74	172.01	143.44	117.49
Std Dev	35.48	23.65	21.40	59.55	43.18	31.55	26.83	20.51	21.43	44.92	31.18	20.26
P-value		<0.0001	<0.0001		<0.0001	<0.0001		<0.0001	<0.0001		<0.0001	<0.0001
PPBG (mg/dL)												
Mean	250.70	201.69	164.96	244.98	183.20	157.46	238.42	203.00	177.47	244.33	191.09	155.60
Std Dev	36.27	27.19	26.92	82.46	40.74	36.28	31.73	29.54	38.24	56.24	35.97	24.21
P-value		<0.0001	<0.0001		<0.0001	<0.0001		<0.0001	<0.0001		<0.0001	<0.0001

## Discussion

The Indian Council of Medical Research (ICMR)-sponsored INDIAB study conducted between 2008 and 2011 revealed a high prevalence of T2DM in India, with an estimated 77 million adults affected according to the International Diabetes Federation (IDF). The study highlighted the substantial burden of cardiovascular risk factors among individuals with diabetes in India, emphasizing the critical need for integrated approaches addressing both diabetes management and cardiovascular health. Assessing the real-world experience of the fixed-dose combination of sitagliptin and dapagliflozin in T2DM patients becomes imperative, given the demonstrated benefits in reducing cardiovascular events and kidney disease progression, as evidenced by studies such as the Canagliflozin and Renal Events in Diabetes with Established Nephropathy Clinical Evaluation (CREDENCE) trial [18-21].

The rationale of the fixed-dose combination of sitagliptin and dapagliflozin in T2DM patients aligns with guidelines from the European Association for the Study of Diabetes (EASD) and the ADA. These guidelines emphasize a multifactorial approach to diabetes management, targeting both glycaemic control and cardiovascular risk reduction. The EASD/European Society of Cardiology (ESC) guidelines recommend pharmacotherapy focusing on agents with proven cardiovascular benefits like SGLT2 inhibitors for patients with T2DM and established cardiovascular disease. Similarly, the ADA advocates for individualized treatment goals and strategies tailored to address cardiovascular risk factors, suggesting the consideration of agents like SGLT2 inhibitors for patients with T2DM and high cardiovascular risk. The fixed-dose combination of sitagliptin and dapagliflozin offers a promising dual therapy approach by combining agents with complementary mechanisms of action and established cardiovascular benefits, potentially optimizing outcomes in T2DM patients, especially those at high cardiovascular risk [4,18].

The combination of sitagliptin and dapagliflozin has been studied in various trials, providing valuable insights into managing T2DM and associated cardiovascular risk factors. The CGMS study demonstrated significant reductions in HbA1c levels and blood glucose without serious adverse events. The DECLARE-TIMI 58 study investigated dapagliflozin's efficacy and safety in patients with T2DM and established or at-risk atherosclerotic cardiovascular disease (ASCVD), showing reduced HbA1c levels and body weight regardless of ASCVD status. It also indicated a decreased risk of cardiovascular events in patients with established ASCVD or multiple risk factors. DECLARE-TIMI 58 substantiates dapagliflozin's efficacy and safety in managing T2DM and associated cardiovascular risk factors [22-24].

In our study, metformin usage was prevalent (85.98%, n=282). Sitagliptin plus dapagliflozin as an adjunct to metformin (1.5 gm/day or equivalent) resulted in reductions in HbA1c, PPBG, and FPG. This aligns with findings from a phase-3 study endorsing the efficacy of dapagliflozin, sitagliptin, and metformin in patients with T2DM poorly controlled with metformin, where it was frequently co-prescribed with the sitagliptin and dapagliflozin fixed-dose combination. Another study comparing dapagliflozin with sitagliptin and metformin in drug-naïve T2DM patients over 12 weeks demonstrated similar beneficial effects on glycaemic parameters. Additionally, a 24-week study revealed that adding dapagliflozin to sitagliptin significantly reduced HbA1c levels compared to placebo, alongside weight reduction and fasting plasma glucose improvements. The clinical evidence supports the efficacy of the sitagliptin and dapagliflozin combination in enhancing key glycaemic parameters and overall patient outcomes [1,4,25].

Considering the role of DPP-4 inhibitors in diabetic hypertension (HTN), our study contributes to the broader understanding of cardiovascular implications associated with T2DM. A review literature suggests that DPP-4 inhibitors may have a positive impact on blood pressure, making them a potential choice in managing hypertensive patients with T2DM. DPP-4 inhibitors prevent the degradation of GLP‐1 and GIP, enhancing insulin secretion and regulating glucose levels. Incorporating DPP-4 inhibitors in the treatment algorithm for T2DM can provide benefits such as improved glycaemic control, reduced inflammation, and potential cardiovascular protection. The co-occurrence of hypertension in the three-fourth majority (n=235, 71.65%) of patients aligns with the findings of a study reporting 67.20% of diabetes mellitus patients exhibiting hypertension [26-28].

TEAE included headaches (6.71%, n=22), changes in micturition (5.49%, n=18), genital mycotic infections (2.13%, n=7), and nausea/diarrhoea (1.83%, n=6). Overall, while dapagliflozin shows a slightly higher incidence of specific adverse events, both medications were generally well tolerated, emphasizing the importance of considering individual patient characteristics and preferences when selecting treatment for type 2 diabetes [29,30].

Several limitations warrant consideration in interpreting the study findings. The sample size may have been insufficient to detect rare adverse events adequately, and the study duration might not fully capture long-term outcomes. The absence of a comparator group or randomization limits direct comparisons. Despite these constraints, the study offers valuable insights into the effectiveness and safety of the sitagliptin and dapagliflozin combination in type 2 diabetes management.

## Conclusions

The present study underscores the significant efficacy of sitagliptin and dapagliflozin fixed-dose combination in achieving glycaemic control, as evident from the significant reduction in HbA1c, FPG and PPBG among Indian T2DM patients. This positive impact extends to individuals with CAD, showcasing the FDC's effectiveness in such population.

Moreover, the findings highlight improvements in blood pressure and lipid profiles, indicating a broad impact on metabolic and cardiovascular parameters. Collectively, these results position the sitagliptin and dapagliflozin FDC as a promising and well-tolerated option for management of T2DM, addressing both glycaemic and cardiovascular aspects.

## References

[1] Bhattacharjee R, Rai M, Joshi P, Prasad A, Birla A (2023). The real DAPSI: a real-world retrospective study on assessing the efficacy and safety of a fixed-dose combination of dapagliflozin and sitagliptin in the Indian population. Cureus.

[2] Singh AK, Sahay R, Gil N (2024). A randomized, double-blind, active-controlled trial assessing the efficacy and safety of a fixed-dose combination (FDC) of metformin hydrochloride 1000 mg ER, sitagliptin phosphate 100 mg, and dapagliflozin propanediol 10 mg in Indian adults with type 2 diabetes: the MESIDA trial. Int J Diabetes Dev Ctries.

[3] Ravikumar L, Kiwalkar RS, Ravindra HS, Lokesh B, Dabhade D (2023). Dapagliflozin and sitagliptin combination therapy: an overview of clinical utility in type 2 diabetes mellitus with multiple cardiovascular risk factors. Cardiol Cardiovasc Med.

[4] Fuchigami A, Shigiyama F, Kitazawa T (2020). Efficacy of dapagliflozin versus sitagliptin on cardiometabolic risk factors in Japanese patients with type 2 diabetes: a prospective, randomized study (DIVERSITY-CVR). Cardiovasc Diabetol.

[5] Inzucchi SE, Bergenstal RM, Buse JB (2015). Management of hyperglycemia in type 2 diabetes, 2015: a patient-centered approach: update to a position statement of the American Diabetes Association and the European Association for the Study of Diabetes. Diabetes Care.

[6] Gajera D, Trivedi V, Thaker P, Rathod M, Dharamsi A (2023). Detailed review on gestational diabetes mellitus with emphasis on pathophysiology, epidemiology, related risk factors, and its subsequent conversion to type 2 diabetes mellitus. Horm Metab Res.

[7] Borse SP, Chhipa AS, Sharma V, Singh DP, Nivsarkar M (2021). Management of type 2 diabetes: current strategies, unfocussed aspects, challenges and alternatives. Med Princ Pract.

[8] Chawla R, Madhu SV, Makkar BM, Ghosh S, Saboo B, Kalra S (2020). RSSDI-ESI clinical practice recommendations for the management of type 2 diabetes mellitus. Indian J Endocrinol Metab.

[9] Scott R, Morgan J, Zimmer Z (2018). A randomized clinical trial of the efficacy and safety of sitagliptin compared with dapagliflozin in patients with type 2 diabetes mellitus and mild renal insufficiency: the CompoSIT-R study. Diabetes Obes Metab.

[10] Saboo B, Agarwal S, Singh AK (2021). Diabetes mellitus and heart failure: a consensus statement. Int J Diabetes Dev Ctries.

[11] Wright EM (2021). SGLT2 inhibitors: physiology and pharmacology. Kidney360.

[12] Jabbour SA, Hardy E, Sugg J, Parikh S (2014). Dapagliflozin is effective as add-on therapy to sitagliptin with or without metformin: a 24-week, multicenter, randomized, double-blind, placebo-controlled study. Diabetes Care.

[13] Dhillon S (2019). Dapagliflozin: a review in type 2 diabetes. Drugs.

[14] Kannan S, Mahadevan S, Ramakrishnan A (2015). Fixed dose combinations for type 2 diabetes. Lancet Diabetes Endocrinol.

[15] Kalra S, Das AK, Priya G (2020). Fixed-dose combination in management of type 2 diabetes mellitus: expert opinion from an international panel. J Family Med Prim Care.

[16] Blonde L, San Juan ZT (2012). Fixed-dose combinations for treatment of type 2 diabetes mellitus. Adv Ther.

[17] Sahay RK, Giri R, Shembalkar JV (2023). Fixed-dose combination of dapagliflozin + sitagliptin + metformin in patients with type 2 diabetes poorly controlled with metformin: phase 3, randomized comparison with dual combinations. Adv Ther.

[18] (2019). IDF Diabetes Atlas 9th ed. https://diabetesatlas.org/upload/resources/material/20200302_133351_IDFATLAS9e-final-web.pdf.

[19] Anjana RM, Deepa M, Pradeepa R (2017). Prevalence of diabetes and prediabetes in 15 states of India: results from the ICMR-INDIAB population-based cross-sectional study. Lancet Diabetes Endocrinol.

[20] Anjana RM, Unnikrishnan R, Deepa M (2023). Metabolic non-communicable disease health report of India: the ICMR-INDIAB national cross-sectional study (ICMR-INDIAB-17). Lancet Diabetes Endocrinol.

[21] Perkovic V, Jardine MJ, Neal B (2019). Canagliflozin and renal outcomes in type 2 diabetes and nephropathy. N Engl J Med.

[22] Breyton AE, Lambert-Porcheron S, Laville M, Vinoy S, Nazare JA (2021). CGMS and glycemic variability, relevance in clinical research to evaluate interventions in T2D, a literature review. Front Endocrinol (Lausanne).

[23] Wiviott SD, Raz I, Bonaca MP (2019). Dapagliflozin and cardiovascular outcomes in type 2 diabetes. N Engl J Med.

[24] Wiviott SD, Raz I, Bonaca MP (2018). The design and rationale for the Dapagliflozin Effect on Cardiovascular Events (DECLARE)-TIMI 58 Trial. Am Heart J.

[25] Ito D, Inoue K, Saito D (2021). Effects of dapagliflozin compared with sitagliptin and metformin in drug-naïve Japanese patients with type 2 diabetes: a 12-week, open-label, randomized, active-controlled trial. Diabetes Ther.

[26] Alsaadon H, Afroz A, Karim A, Habib SH, Alramadan MJ, Billah B, Shetty AN (2022). Hypertension and its related factors among patients with type 2 diabetes mellitus - a multi-hospital study in Bangladesh. BMC Public Health.

[27] Yin R, Xu Y, Wang X, Yang L, Zhao D (2022). Role of dipeptidyl peptidase 4 inhibitors in antidiabetic treatment. Molecules.

[28] Saini K, Sharma S, Khan Y (2023). DPP-4 inhibitors for treating T2DM - hype or hope? an analysis based on the current literature. Front Mol Biosci.

[29] Sun Y, Yan D, Hao Z, Cui L, Li G (2020). Effects of dapagliflozin and sitagliptin on insulin resistant and body fat distribution in newly diagnosed type 2 diabetic patients. Med Sci Monit.

[30] Raji A, Xu ZJ, Lam RL, O'Neill EA, Kaufman KD, Engel SS (2020). Efficacy and safety of sitagliptin compared with dapagliflozin in people ≥ 65 years old with type 2 diabetes and mild renal insufficiency. Diabetes Ther.

